# Assessing Salivary Ductal Structures of the Donkey (*Equus asinus*) Using Conventional Sialography and Its Practical Guide

**DOI:** 10.1155/vmi/9920803

**Published:** 2025-10-09

**Authors:** Jamal Nourinezhad, Albert Abdi, Abdolvahed Moarabi, Mohammad Ghasem Hanafi, Rahmat Allah Fatahin Dehkordi, Anna Tomańska

**Affiliations:** ^1^Division of Anatomy and Embryology, Department of Basic Sciences, Faculty of Veterinary Medicine, Shahid Chamran University of Ahvaz, Ahvaz, Iran; ^2^Department of Basic Sciences, Faculty of Veterinary Medicine, Shahid Chamran University of Ahvaz, Ahvaz, Iran; ^3^Division of Radiology, Department of Clinical Sciences, Faculty of Veterinary Medicine, Shahid Chamran University of Ahvaz, Ahvaz, Iran; ^4^Department of Radiology, Ahvaz Jundishapur University of Medical Sciences, Ahvaz, Iran; ^5^Department of Basic Sciences, Faculty of Veterinary Medicine, Shahrekord University, Shahrekord, Iran; ^6^Division of Animal Anatomy, Department of Biostructure and Animal Physiology, Faculty of Veterinary Medicine, Wroclaw University of Environmental and Life Sciences, Wrocław, Poland

**Keywords:** anatomy, equine, salivary, salivary diseases, salivary ducts, sialography

## Abstract

Sialoradiography is an imaging technique that assesses the ducal macroanatomy and function of the major salivary glands (MSGs). Despite reports of salivary diseases in donkeys, no studies have documented detailed information on sialoradiography of the ductal structures. Therefore, this investigation aimed at describing the technique and ductal macroanatomy of the MSGs using sialoradiography, as well as the *in situ* macroanatomy of the salivary ductal structures in donkeys. Survey radiology and sialoradiography were conducted in lateral and dorsoventral oblique views, followed by cadaver dissection with colored latex injection to outline the MSGs' topographic macroanatomy in fourteen half-heads and the proximal cervical region of seven donkeys. The technique of cannulation, contrast media injection, and positioning for the sialoradiography in the donkey was thoroughly described. The extra- and intraglandular salivary ductal structures of the parotid and mandibular glands were clearly outlined only in lateral sialograms. Key macoanatomical findings included the rostral situation of the mandibular gland, a straight caudal edge of the parotid salivary gland (PSG), the absence of perforation of the PSG lateral aspect and its substance by the maxillary vein, and the superficial situation of the parotid duct (PD) on the body of the lower jaw rostral to in front of the masseter muscle, situation of the PD opening against the crowns of the third and fourth premolar teeth, and the formation of the single major PD at the rostrolateral aspect of the dorsal portion of PSG. The images and data reported in the current investigation may be utilized as basic information for veterinary clinicians, surgeons, and radiologists for (1) diagnosis of the MSGs pathologies and (2) aiding in performing sialography in live donkeys.

## 1. Introduction

The donkey and domestic horse share a common ancestry but have anatomical, genetic, and physiological differences, many of which affect their management, welfare, clinical examination, surgery, and medicine. An increase in the use of donkeys for the production of meat, milk, companions, and continued use as pack animals [1–3], as well as nature conservation, tourism, and the cosmetic industry [4], have increased the interest and value in this species. New findings focused on various subjects of clinical and basic sciences, although a significant rise in diagnostic imaging and macroanatomy investigations in donkeys has been highlighted [5–13].

The major salivary glands (MSGs) in herbivores, which include the parotid, mandibular, and sublingual glands, secrete saliva into the oral cavity [14]. Salivary glands and their secretions play a significant role in preserving the health of oral structures and the gastric environment [15]. Consequently, conditions affecting the salivary glands and their function directly impact teeth, oral mucosa, and stomach [16].

Diseases of the salivary glands and ducts, including sialadenitis, salivary calculi, salivary mucocele, trauma, and neoplasia, are uncommon in horses [16]. Sialolithiasis occurs more frequently in equine species than in other domestic species and is the most common condition to affect the salivary ducts and, much less commonly, the glands themselves [17–20]. The incidence of sialolithiasis is higher in arid environments, and the prevalence of sialoliths in donkeys is notably elevated, with a 70% occurrence, compared to other equids, where horses have a 20% occurrence and mules have a 10% occurrence. The etiology of sialolith formation remains unknown. Whether this discrepancy is reflected in ductal anatomy, saliva composition, quantity, or dietary factors is a question that warrants further investigation [18]. And the first and essential step to answer this question is to thoroughly understand the typical ductal macroanatomy of MSGs in the donkey.

Sialoradiography is a radiographic technique where a radiopaque contrast medium is infused into the ductal structures of a salivary gland before imaging with plain films/digital image receptors and computed tomography (CT). Sialoradiography is the most detailed way to outline the ductal structures [21]. Sialoradiography is a diagnostic test for the evaluation of the ducal pathoses [16, 20, 22] in presurgical [16, 22–24] or postsurgical [25] planning. Typical subtle macroanatomy of the salivary ductal structures has been reported using sialoradiography in cows, dromedaries, ovine, horses [26–29], and caprine [30]. To accurately evaluate and interpret sialograms, an accurate and complete knowledge of the salivary ductal structures of macroanatomy is absolutely necessary [31]. Nevertheless, a literature review revealed that such reports have not been made on donkeys.

Therefore, in line with our research on the branching ducal pattern of water buffaloes in sialoradiography and *in situ* macroanatomy [32]. The current investigation was conducted to achieve the following objectives: (1) to, for the first time, delineate the fine ductal structures of the donkey MSGs using the sialoradiography technique; (2) to record the technique used to acquire a typical MSGs sialogram; (3) to present detailed morpho-topographic accounts of the donkey ductal structures *in situ*; and (4) to compare our sialoradiography and macroanatomical findings with those of horses and wild and domestic ruminants.

## 2. Materials and Methods

### 2.1. Examined Animals

Fourteen half heads and the proximal cervical area of seven adult donkeys (four males and three females), aging 4–6 years, were used in this investigation. The specimens were intact, without any external abnormalities or pathologies. The investigation was conducted in compliance with Local Ethical Committee Guidelines for animal experiments. The animals were euthanized based on American Veterinary Medical Association guidelines [33] to immediately carry out the survey radiography, sialoradiography, and macroanatomy.

### 2.2. Survey Radiography

Scout films in the lateral oblique and ventrodorsal oblique projections were acquired using a Toshiba KCD-10M-6AIT portable X-ray unit (Japan) and System: Prima CR radiology device (CR-I392 model, Fujifilm, Japan) with an output of 65 kV and 300 mA before infusing the contrast solution into the ductal structures to grossly detect radiopaque sialolithiasis or identify any abnormalities.

### 2.3. Sialoradiography of the Parotid Duct (PD)

The mouth cavity was opened using a mouth gag, but care must be taken not to open the jaw so wide that the buccal mucosa is under tension, making the cannulization challenging. After drying the mucosa with gauze, the PD opening is identified in the buccal mucosa against the maxillary second cheek teeth. Because of the small parotid papilla, the identification of the opening was challenging. For this reason, a droplet of saliva can be seen at the opening of the PD with careful massage of the parotid salivary gland (PSG).

Once the opening is identified, the dilators can be utilized to widen the opening for easier cannula placement. The abduction of the cheek with the thumb and index finger often provided better exposure and a better angle for cannula insertion. Headlight unit magnifying spectacles were applied during the catheterization of the PD. A small feeding tube was utilized to prevent laceration of the papillae and perforation of the PD wall. Then, the cannula was gently inserted, and a water-soluble contrast material (Visipaque, GE Healthcare, Oslo, Norway) was slowly injected. The sialoradiography was done in two phases: (1) duct filling, which was conducted to outline the ductal arborization of the PSG. By suboptimal filling of the ductal structures in this phase, some areas of the ductal structures of the gland may not be seen. (2) Acinar or parenchymatous filling was conducted to observe the acini. Only 1 mL of a water-soluble contrast material should be injected gently for the ducal filling by the pressure of the hand. Only 1.5 mL of the contrast material should be injected for the acinar filling. By overfilling the ductal structures and a complete acinarization of the PSG, the detailed intraglandular ducts and the architecture of branches were obscured entirely, rendering the evaluation virtually useless. Care must be taken not to obscure the ductal macroanatomy.

### 2.4. Sialoradiography of the Mandibular Duct (MD)

To best outline the openings of the MD, the area should be dried with gauze, and the tongue should be pushed outside through a wide interdental space or diastema to expose the sublingual caruncles and to put some tension on the caruncles. Milking the mandibular gland was done to aid in identifying the opening by producing a drop of saliva. The opening was smaller than that of the PSG. Thus, it was usually more difficult to catheterize the MD than the PD. The caruncle should be raised and steadied to catheterize the MD by grasping it with fine, smooth forceps. To better outline the opening of the MD, the magnifier should be utilized during MD catheterization. Gentle rubbing of the peripheral venous catheter (gauge: 18; green color) in a rostrocaudal direction with a slight rotation can help the cannula to enter and dilate the opening. Only 0.2–0.5 mL of the contrast material should be injected for ductal filling. Only 0.5–1 mL of the contrast material should be injected for acinar filling.

#### 2.4.1. Sialographic Projection

After each phase, lateral oblique and dorsoventral oblique radiographs of the head and proximal neck were immediately made with a 35 × 35 cassette situated under the side being evaluated and projected from the contralateral side of the head while the syringe was still connected. Once the ducts were catheterized, care must be taken not to dislodge them during imaging, as this could lead to opacification of the area.

### 2.5. Macroanatomy

MSGs with their ducts and adjacent structures were immediately dissected layer by layer in fresh donkey heads after imaging. Heads were sectioned along the median line using an electric saw to investigate ductal macroanatomy of MSGs. Dissections under a magnifier (LTS 120, China, 2.5x) were enhanced by injecting red (Sahar colour, Tehran, Iran) colored latex (1-Stop Solution, Kuala Lumpur, Malaysia) into the carotid artery to highlight nerves, veins, and ducts. Specimens were preserved in 10% formaldehyde, and photographs with a steel ruler for the scale were taken using an iPhone 13 Pro Max. For the nomenclature, N.A.V. [34] was considered.

## 3. Results

No clinical complications were observed in any of the examined heads. The MSGs sialoradiography and topographic macroanatomy did not exhibit sex differences and laterality. The topographic macroanatomy of the MSG ductal structures is exhibited in detail (Figures 1, 2, 3, 4, 5, 6, 7, 8). The survey radiography of the dorsoventral and lateral views of the head is exhibited (Figures 9 and 10). The parotid and mandibular sialograms of the examined donkeys are shown in Figures 11, 12, 13, 14, 15.

### 3.1. Macroanatomy

#### 3.1.1. PSG (Figure 1)

**Figure 1:**
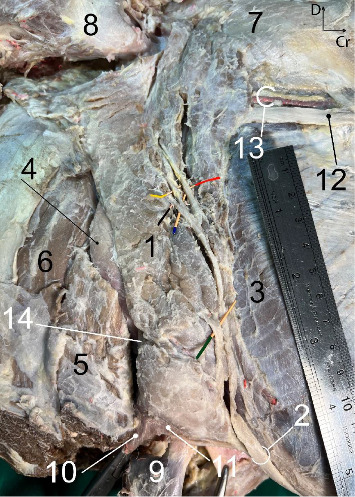
The right parotid salivary gland in the six-year-old male donkey, *in situ*: lateral view. Four radicles of the parotid duct (indicated with various angiocath colors). The parotidoauricularis muscle has been removed to display the gland. A portion of the insertion aponeurosis of the cleidomastoideus has also been excised to reveal the mandibular salivary gland and the obliquus capitis cranialis muscle. 1. Parotid gland, 2. Parotid duct, 3. Masseteric muscle, 4. Mandibular salivary gland, 5. Cleidomastoideus, 6. Obliquus capitis cranialis muscle, 7. Temporalis, 8. Auricular cartilage, 9. Insertion tendon of the sternomandibularis, 10. External jugular vein, 11. Linguofacial vein, 12. Facial nerve, 13. Transverse facial vessels, 14. Maxillary vein, Cr. Cranial, D. Dorsal. Scale: 1 mm.

The PSG appeared as a clearly lobulated structure, being the largest salivary gland and forming a compact organ. The gland exhibited a flat and thick appearance with a light yellowish color in a fresh state. It was almost long and roughly rectangular in shape and occupied the entire retromandibular fossa (Figure 1).

**Figure 2:**
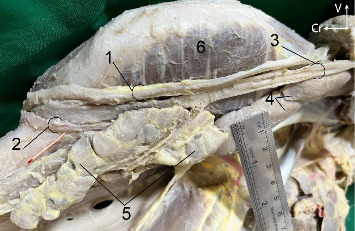
The route and topography of the mandibular portion of the left parotid duct in the six-year-old male donkey, *in situ*: ventroventromedial view. The larynx and pharynx have been displaced dorsally to provide a clearer presentation of additional structures. 1. Parotid duct (catheter in place), 2. Facial artery, 3. Lingofacial vein, 4. Mandibular salivary gland, 5. Mandibular lymph node, 6. Medial pterygoid muscle, Cr. Cranial, V. Ventral. Scale: 1 mm.

The ventral narrow end was situated at the angle between the maxillary and linguofacial veins and covered almost the veins (Figure 1). The thick dorsal end is notched to fit the base of the ear (Figure 2). The thin rostral border of the gland was largely level with the masseter muscle. It is closely attached to the caudal border of the mandibular ramus and masseter muscle, except for a small flat area of the gland that extended some distance over the masseter directly. The border was closely attached to the caudal border of the mandibular ramus (Figure 1). The caudal border was almost straight. It was related to the cleidomastoideus, the obliquus capitis caudalis, and the longus capitis muscles (Figure 1).

The uneven lateral aspect of the PSG was partially covered by the parotidoauricularis muscle and parotid fascia (Figure 1). The uneven medial aspect of the PSG was related to the occipitomandibular belly of the digastricus, occipitohyoideus, obliquus capitis cranialis muscles, caudal lobe of the mandibular salivary gland, the guttural pouch, and insertion tendon of the sternomandibular muscle (not shown). The maxillary vein of the donkey initially followed the caudal border of the medial aspect of the PSG, and then crossed obliquely over the medial aspect of the gland to reach the caudal border of the mandibular ramus without perforating the lateral aspect and gland substance. The vein formed a deep groove over the medial aspect of the gland (Figure 1).

#### 3.1.2. The Origin, Course, and Termination of the PD

**Figure 3:**
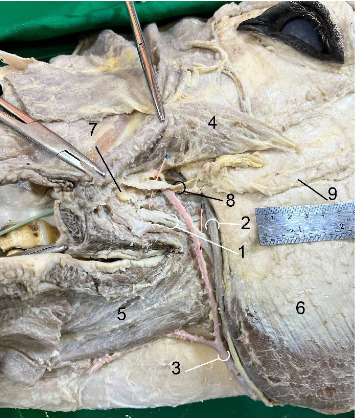
The facial portion of the left parotid duct in the six-year-old male donkey, *in situ*: lateral view. The zygomatic muscle has been cut and reflected caudally to demonstrate the parotid duct and adjacent vessels and nerves. 1. Parotid duct (catheter in place), 2. Facial vein, 3. Facial artery, 4. Zygomaticus, 5. Buccinator muscle, 6. Masseteric muscle, 7. Dorsal buccal salivary gland, 8. Dorsal buccal nerve, 9. Facial nerve. Scale: 1 mm.

**Figure 4:**
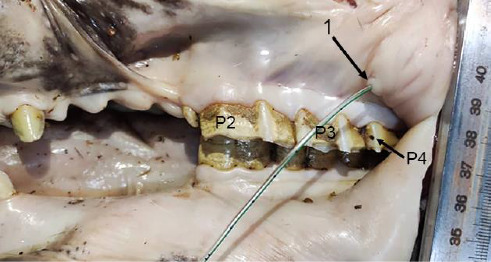
The six-year-old male donkey's left parotid opening (catheter in place), rostrolateral view: 1. Parotid papilla (arrow), P2-4. Maxillary second to fourth premolar teeth. Scale: 1 mm.

The single duct was formed by the union of the three sizeable radicles (branch roots) at the lateral aspect close to the rostral border of the proximal part of the gland. One radicle joined the single duct at the distal part of the rostrolateral aspect of the gland. After that, the main duct first ran along the rostral border of the gland (Figure 1) and then coursed on the medial aspect of the mandible over the medial pterygoid muscle, accompanying the facial vein and artery. It then wound around the ventral border of the mandibular body to reach the lateral aspect of the face (Figure 2). Over the lateral aspect of the mandibular body, just rostral to the masseter muscle and before reaching the buccinator muscle, the most rostral vessel was the facial artery. Caudal to the artery was the PD, and caudal to the PD was the vein, making the PD superficial. Subsequently, the duct passed rostrodorsally beneath the facial vessels and buccal nerve to open in the space between the buccinator muscle and the lateral wall of the vestibule, penetrating the dorsal buccal salivary gland. In this situation, the duct was laterally covered by the insertion of the zygomatic muscle (Figure 3). The duct terminated by opening into the buccal vestibule at a distinct parotid papilla, opposite the crowns of the third and fourth premolar teeth (Figure 4). The opening of the PD was larger than that of the MD (Figure 8).

#### 3.1.3. MSG (Figure 5)

The MSG was smaller than the PSG and resembled a boomerang in shape. The coarsely lobulated gland had a yellowish color in a fresh state. Its parenchyma was thickly encapsulated by collagenous tissue, and it possessed the thickest capsule among the glands. The lobules of MSG did not share the same dimensions as the parotid lobules and were loosely connected with fewer intervening connective tissues. MSG lobules were larger than those of the PSG. The MSG was extended from the wing of the atlas to the medial aspect of the mandibular ramus, near the basihyoid bone, at the level of the caudal end of the mylohyoid muscle. Due to its deep situation, MSG was not palpable. It was divided into the caudal lobe and the rostral lobe. Nevertheless, they were surrounded by a common capsule and separable at the level of the ventral end of the occipitomandibular belly of the digastricus muscle. The MSG is separated laterally from the PSG by the maxillary vein and the insertion tendon of the sternomandibular muscle.

##### 3.1.3.1. Caudal Lobe (Figure 5)

The oval caudal lobe was thicker and larger than the rostral part, extending from the wing of the atlas to the caudal intermandibular space. The lateral aspect of the lobe was almost entirely covered by the PSG. Two-thirds of the aspect was concealed by the mandibular ramus, while one-third was covered by the PSG. A small portion of the caudal lobe extended caudally beyond the caudal border of the PSG, being concealed by the parotid fascia and an amount of connective and fat tissue (Figure 1). The aspect was in close relation to the occipitomandibular belly of the digastricus muscle, the PSG, the maxillary vein, and the insertion tendon of the sterno-mandibular muscle (not shown). The medial aspect was surrounded by a substantial amount of connective tissue, the guttural pouch, and the lateral retropharyngeal lymph node (not shown). The narrow and rounded dorsal or proximal end was in contact with the longus capitis muscle and medial compartment of the guttural pouch. This ventrorostral end was in relation with the common carotid artery, vago-sympathetic trunk, cranial cervical lymph node, insertion tendon of the sternomandibular muscle, thyroid and cricoid cartilages, and cricothyroid muscle. The rostral border was flat but slightly concave in its middle. The caudal border was free, convex, and thick, protruding from the caudal edge of the PSG.

##### 3.1.3.2. Rostral Lobe (Figure 5)

This part was located entirely medial to the ramus of the mandible. The flat lateral aspect of the lobe was in contact with the medial pterygoid muscle and ventral end of the occipitomandibular belly of the digastricus muscles. The convex and uneven medial aspect of the lobe was covered by the larynx and a quantity of connective tissue. This rostral rounded end was related to the lobulated mandibular lymph node. The caudal end of the lobe was in contact with the caudal lobe. The concave dorsal border was attached to the occipitomandibular belly by connective tissue. The ventral convex border of the lobe was in contact with the linguofacial vein and thyroid cartilage.

##### 3.1.3.3. The Origin, Course, and Termination of the MD

**Figure 5:**
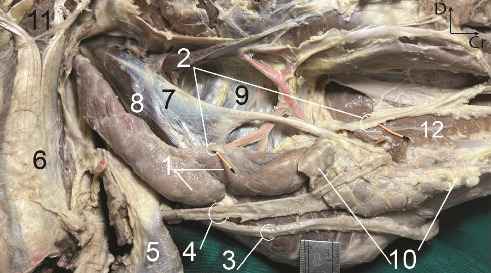
Sagittal section of the head and proximal region of the neck in the six-year-old male donkey showing the pterygomandibular region, *in situ*: on the left side, the larynx, pharynx, and associated muscles have been displaced dorsally to show the gland and its associations. 1. Mandibular salivary gland, 2. Mandibular duct, 3. Parotid duct (catheter in place), 4. Facial vein, 5. Sternomandibularis, 6. Longus colli muscle, 7. Caudal portion of the digastricus, 8. Occipito-mandibular portion of the digastricus, 9. Medial pterygoideus, 10. Mandibular lymph node, 11. Longus capitis muscle, 12. Mylohyoideus, Cr. Cranial, D. Dorsal. Scale: 1 mm.

**Figure 6:**
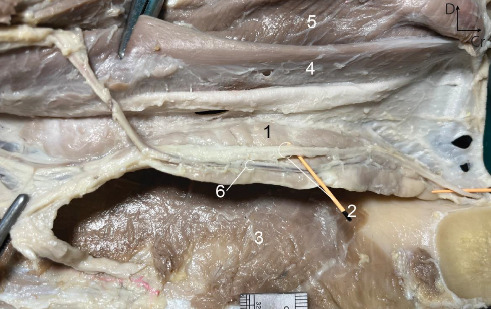
The left polystomatic sublingual salivary gland in the six-year-old male donkey, *in situ*: medial view. 1. Polystomatic sublingual salivary gland, 2. Mandibular duct, 3. Mylohyoideus, 4. Geniohyoideus, 5. Genioglossus, 6. Lingual artery, Cr. Cranial, D: Dorsal. Scale: 1 mm.

**Figure 7:**
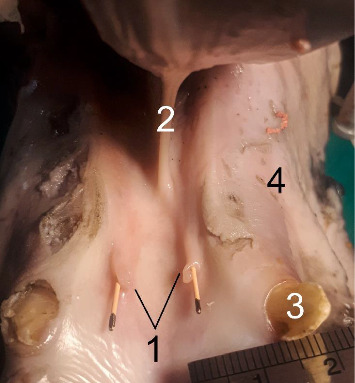
Sublingual floor of the oral cavity in the male six-year-old donkey. Openings of the mandibular ducts (black angiocaths). 1. Sublingual caruncles, 2. Frenulum lingua, 3. Canine teeth, 4. Diastoma. Scale: 1 mm.

The long and narrow duct was formed by a single large radicle (branch) originating from the rostral end of the caudo-dorsal part of the gland near its medial aspect. The duct then passed over the MSG for a short distance, continuing over the medial aspect of the occipitomandibular belly of the digastricus muscle. After that, the duct coursed along the dorsal border of the rostral gland over the medial aspect of the medial pterygoid muscle. Along this course, the duct was covered by the larynx. The duct crossed the intermediate tendon of the digastricus muscle after leaving the rostral extremity. It proceeded between the hyoglossus and mylohyoid muscles for a very short distance (Figure 5). The duct extended rostrally between the mylohyoid and geniohyoid muscles. Further rostrally, the duct passed over the middle of the medial aspect of the rostral extremity of the polystomatic sublingual gland (Figure 6) and opened independently just caudal to the level of the canine to the small orifice on the ventral aspect of the narrow and flat sublingual caruncles, near the midline (Figure 7).

### 3.2. Sublingual Salivary Glands (SGs)

#### 3.2.1. Monostomatic SGs

This type of SGs was absent.

#### 3.2.2. Polystomatic SGs (Figures 6 and 8)

**Figure 8:**
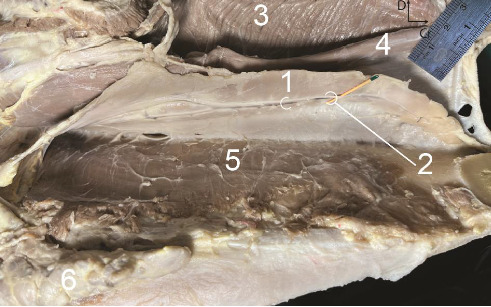
The left polystomatic sublingual salivary gland in the six-year-old male donkey, *in situ*: Lateral view. 1. Polystomatic sublingual salivary gland, 2. Lingual nerve and vein, 3. Genioglossus muscle, 4. Geniohyoideus, 5. Mylohyoideus, 6. Mandibular lymph node, Cr. Cranial, D. Dorsal. Scale: 1 mm.

The lobulated gland was fusiform and laterally flattened, extending from the caudal part of the mandibular symphysis to the level of the second molar tooth (M2). The rostral end was narrow and thin, directly related to the incisive part of the mandible. The caudal end was smaller than the rostral end and related to the lingual nerve. The ventral border was thicker than the dorsal border and was not related to ducts and vessels, but the branches of the lingual nerve coursed rostrally along this border. The lateral aspect was flat and covered by the mylohyoid muscle, except for its rostral extremity (Figure 8). The lingual nerve and vein coursed over the aspect (Figure 8). The medial aspect was slightly convex. One-third of the rostral part of the aspect was in contact with the genioglossus muscle, while two-thirds of the caudal part of the gland was related to the styloglossus muscle. The MD and lingual artery coursed over the rostral half of this aspect near the dorsal border (Figure 6). The gland possessed numerous small ductules, each terminated by opening into the lateral sublingual recess (not shown).

### 3.3. Sialoradiography

**Figure 9:**
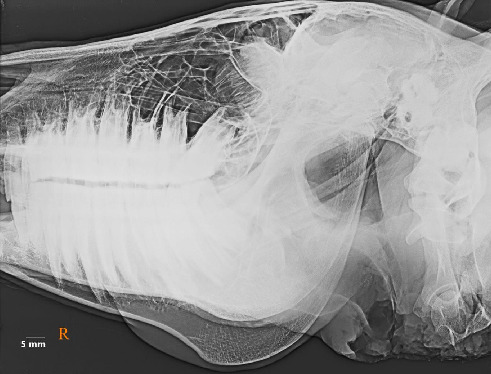
Lateral oblique survey radiography of the head and neck in the five-year-old female donkey outlining none of the left major salivary glands. Scale bar: 5 mm.

**Figure 10:**
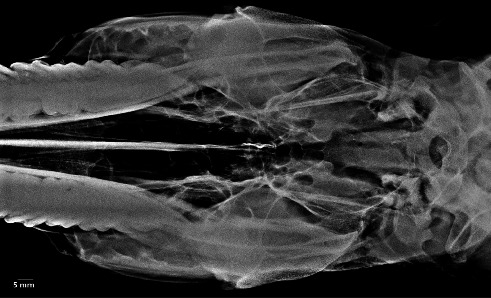
Dorsoventral oblique survey radiography of the head and neck in the five-year-old female donkey outlining none of the major salivary glands. Scale bar: 5 mm.

**Figure 11:**
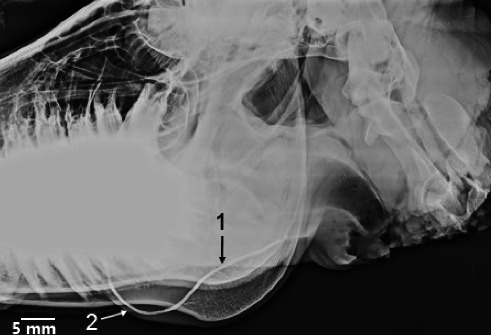
Lateral oblique sialogram of the left parotid gland in the five-year-old female donkey depicting the ductal opacification phase. 1. Major duct, 2. Curvature (bending). Scale bar: 5 mm.

**Figure 12:**
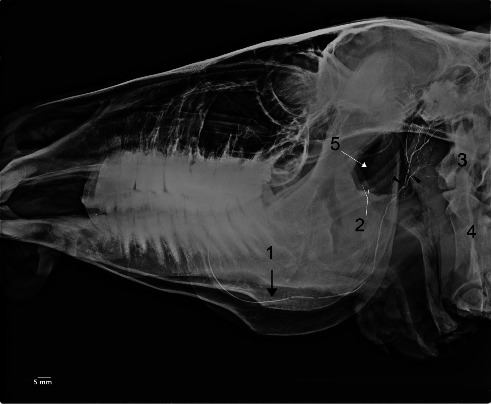
Lateral oblique sialogram of the left parotid gland in the five-year-old female donkey demonstrating the acinar opacification phase. Several radicles (arrows) and fine branches have been outlined. 1. Major duct, 2. Stylohyoid bones, 3. Atlas, 4, Axis, 5. Guttural pouch. Scale bar: 5 mm.

**Figure 13:**
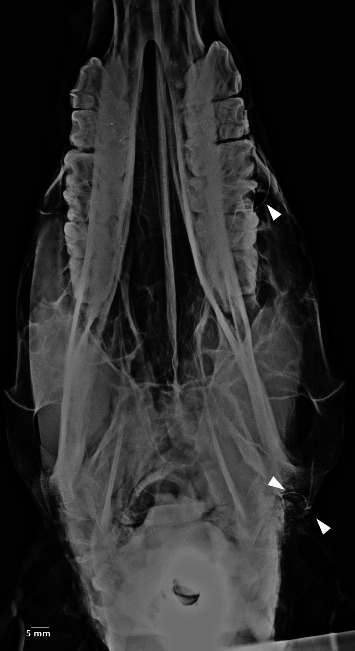
Dorsoventral oblique sialogram of the left parotid gland in the five-year-old female donkey. The fine ductal macroanatomy (arrows) has not been outlined due to superimposition by the bony structures of the skull, atlas, and axis. Scale bar: 5 mm.

**Figure 14:**
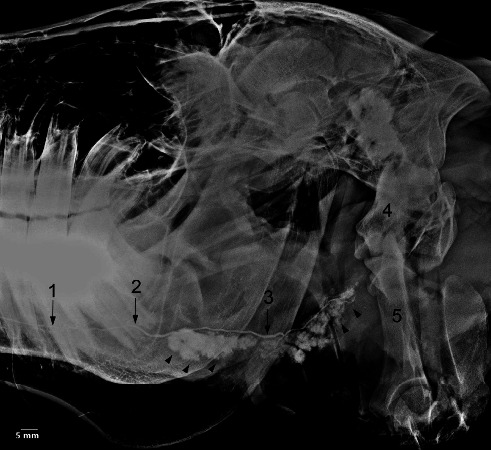
Lateral oblique sialogram of the left mandibular gland in the five-year-old female donkey showing the acinar opacification phase. The outline of the gland body is discernible (arrows). 1. Major duct, 2. Curvature (bending), 3. Radicle, 4. Atlas, 5. Axis. The peripheral intramandibular ductal structures have been outlined. Scale bar: 5 mm.

**Figure 15:**
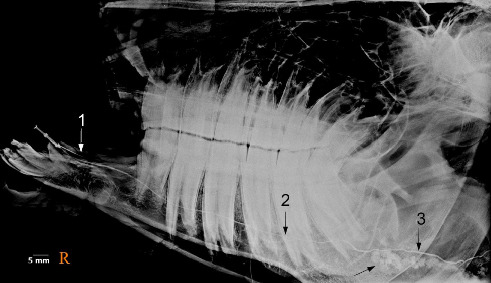
Lateral oblique sialogram of the left mandibular gland in the five-year-old female donkey revealing acinar filling. The outline of some portion of the gland body is discernible (black arrow). 1. The opening of the mandibular duct (catheter in place, white arrow), 2. Major duct, 3. Radicle. Scale bar: 5 mm.

The MSGs and their ducts were not outlined on the lateral oblique or dorsoventral oblique survey radiographs (Figures 9 and 10). The fine macroanatomy of the ductal structures in the PSG and MSG was clearly visualized in the lateral oblique sialograms (Figures 11, 12, 14, and 15), and the specimens displayed completely typical sialogram appearances. The fine ductal macroanatomy of the PSG (Figure 13) or MSG (not shown) was not outlined on dorsoventral oblique sialograms due to superimposition by the bony structures of the skull, the atlas, and the axis. Bilateral sialoradiography of the PSG or MSG resulted in sialogram failure due to superimposition by the ductal structures (not shown). The intraglandular ductal conformation of the PSG and MSG exhibited an arborizing or tree-like branching pattern on the lateral oblique sialograms (Figures 12 and 14). Nevertheless, the intraglandular ductal structures differed between the PSG and MSG. To clarify, the central and peripheral intraglandular ductal structures were distinct.

#### 3.3.1. Sialoradiography of the PSG

The ductal opacification of the PSG was depicted on the lateral oblique sialograms (11). The acinar opacification of the PSG, featuring two clearly visible large radicles and several fine branches (ductules), was visualized in the lateral oblique sialogram (Figure 12). The orifice of the PD is obscured due to the high density of the upper cheek teeth (Figures 11 and 12). The single main PD (extraglandular duct) was formed in the retromandibular space at the level of the middle of the caudal border of the mandibular ramus through the union of several large radicles (branches) (Figure 12). The intraparotid ductal superimposed over the proximal part of the stylohyoid bone and the large radiolucent guttural pouch, extending caudally to the level of the ventral aspect of the atlantoaxial joint (Figure 12). The primary PD appeared as a curve with the convexity ventral (Figure 12). The opacity of the terminal part of the main PD rostral to the masseter muscle was considerably attenuated due to compression by the muscle and the high density of the mandible and cheek teeth (Figures 11 and 12). Fine branches originated from the large radicles of the PSG in various directions (Figure 12).

#### 3.3.2. Sialoradiography of the MSG

The acinar opacification throughout the MSG, featuring a clearly visible large radicle and several fine branches (ductules), was visualized in the lateral oblique sialogram (Figures 14 and 15). The main MD was commenced from the rostral border of the dorsal end of the gland at the level of the ventral aspect of the atlantoaxial joint in the retromandibular space. It then passed rostrally to the distal part of the mandibular ramus. It proceeded rostrally parallel to the middle of the molar part of the mandibular body, crossing the distal part of the cheek teeth. After that, at the level of the diastema, it extended parallel to the interalveolar border of the incisive part of the mandibular body to its opening (Figures 14 and 15). At the level of the root of the third lower molar tooth, the primary duct, just before reaching the gland, formed a curvature (bending) opening ventrally (Figures 14 and 15). Fine branches originated only from the ventral aspect of the large radicle of the MSG (Figure 14). The peripheral branches of the intraglandular ductal structures in the MSG were much shorter and tapered more abruptly than those in the PSG (Figure 14).

## 4. Discussion

### 4.1. Macroanatomy

Maher et al. [35] dissected the infra-auricular parotid region of the donkey. They provided very few anatomical accounts about the PSG and MSG, showing only the lateral aspect of the PSG. Furthermore, Shaker and Abood [36] documented little data on the situation, relation, shape, size, and weight of the PSG and MSG of the male donkey and exhibited few photographs. The above-mentioned studies did not describe the formation, route, ending, and relation of the salivary ducal structures. Nevertheless, for the first time, the current investigation provided detailed, colorful descriptions of the topographic macroanatomy of MSGs and their ductal structures in the donkey. We also documented comprehensive descriptions on the comparative macroanatomy of the MSGs among donkeys, horses, dromedaries, domestic, and wild ruminants.

#### 4.1.1. PSG Shape

The shape of the PSG in herbivores varies significantly. In ovine [31], mouflon, and deer [37], dromedary [38], donkey [35, 36], and horse [39–41], it typically occurs as a long rectangular structure shape, resembling the current investigation. Nevertheless, in the cow and water buffaloes, the PSG takes on a club-shaped form [32, 42] or appears elongated [40].

#### 4.1.2. PSG Location and Relation

The PSG displays situational variations among different species. As in the mouflon and deer [37], deer [43, 44], dromedary [38], ovine [45], and horse [39–41], the PSG in the donkey completely fills the retromandibular fossa. Nevertheless, the PSG does not fill the fossa in the cow and water buffaloes [31, 32].

In the horse, the lateral aspect of the PSG in the horse is obliquely crossed by a vein, which is largely embedded in the PSG [14, 31, 39, 42, 46, 47]. Additionally, the maxillary vein of the horse initially follows the caudal edge of the PSG, then perforates the lateral aspect of the PSG and passes through the PSG at the caudal edge of the ramus of the lower jaw [42].

In contrast, in the current investigation, the vein passes medial to the PSG without perforating the lateral aspect of the PSG, resembling domestic ruminants [31, 46], donkeys [48], and dromedaries [38].

The caudal edge of the PSG in the examined donkey is almost straight, resembling the donkey [35]. Nevertheless, in the horse, the edge is concave [39, 42]; it extends to the wing of the first cervical vertebra [23], while in the donkey, the caudal edge extends to the body of the axis.

In contrast to the examined donkeys, the ventral extremity of the PSG is wider than the dorsal extremity in horses, and the maxillary and linguofacial veins are not covered by the ventral extremity of the PSG [39, 42].

#### 4.1.3. The Beginning, Route, and Ending of the PD

The PD is made by the joining of two radicles in the mouflon, deer [37], pampas deer [43], and water buffaloes [32]. In contrast, the duct is made by the convergence of three to four radicles in the dromedary [49] and horse [14, 31, 39–41].

The single major duct is made at the medial aspect of the middle portion of the ventral extremity of the PSG in the cow [31] and water buffaloes [32]. In horses, the single major duct is made at the rostrolateral aspect of the ventral portion of the PSG [31], or the mandibular angle, specifically the rostroventral angle [14, 23, 42, 46, 47]. In our investigation, the single major duct is made at the lateral aspect, very near the rostral edge of the proximal portion of the PSG, which disagrees with the reported findings in the horse. It is worth noting that the confluence of the radicles to form the major duct in horses has shown situational variations between the authors of English and non-English anatomical books. Therefore, further analysis is essential to determine the exact situation of the joining of the radicles.

From an embryological viewpoint, MSGs arise through the ingrowth of oral epithelium into underlying mesenchyme. The PSG and MSG are unique in that they migrate during development, resulting in their acquisition of long ducts for saliva transmission [50]. This migration accounts for the variable situations of the duct openings in different mammalian taxa. For instance, the opening of the PD in the oral vestibule is quite variable among herbivores. It opens against the P2 tooth in giraffes [51] and the P3 in horses [16, 40, 41]. In some cases, it opens against the P4 tooth in horses [14, 40–42, 52], the M1 in the dromedary [38], the M2 tooth in the cow [50], and the M3 in the horse [39], or different teeth in various species such as the ovine [42], mouflon, and deer [37, 43, 44], and water buffaloes [32]. In the current investigation, the PD opens against the crowns of the P3 and P4, inconsistent with the reported results in horses. Nevertheless, it is important to note that descriptions of the PD opening in horses vary among different authors, and these discrepancies emphasize the need for a re-analysis of the PD opening to determine its exact situation.

The route of the PD exhibits variations among herbivore species. In most ruminants, including deer and giraffes [37, 43, 51], water buffaloes [32], cows [52], dromedaries [38], the mandibular portion of the PD follows the ventral and rostral edge of the masseter muscle. In contrast, in ovine, caprine, and mouflon [37], the PD crosses the lateral aspect of the masseter muscle. Nevertheless, in donkeys and horses, the PD runs initially on the medial aspect of the lower jaw after leaving the PSG. It then winds around the ventral edge of the body of the lower jaw to reach the lateral aspect of the face, resembling the route observed in horses [31, 41, 42]. Therefore, it can be concluded that the mandibular portion of PD in donkeys and horses, in contrast to most ruminants and dromedaries, is a valuable anatomical distinction.

The interesting difference between the syntropy of the PD and facial vessels on the lateral aspect of the body of the lower jaw before reaching the buccinator muscle in horses and donkeys is noteworthy. In donkeys, the most rostral vessel in this region is the facial artery, followed by the PD, and then the vein. As a result, this portion of the PD is superficial and relatively vulnerable. Nevertheless, in horses, the configuration is distinct. Caudal to the artery lies the facial vein, and the PD is situated caudal to the vein and is covered by the vein and the rostral margin of the masseter muscle [14, 31, 39, 40, 42, 52]. In this situation, the PD in donkeys is prominently discernible, whereas in horses, the PD is comparatively exposed primarily toward the extremity of its route [14]. In a clinical context, Dixon and du Toit [53] noted that the caudal segments of the PD in horses might be at risk of damage during cheek teeth repulsion, while the PD proves to be more valuable in its rostral sections when considering cheek teeth extraction and lateral buccotomy techniques.

On the other hand, the most common disorder affecting the MSGs in horses is trauma to the PSG or its duct. Due to their superficial situation, they are predisposed to injury. Signs of damage to the gland or duct include cutaneous damage in the area where the PSG is positioned or to the tissue at the caudal or ventral edge of the lower jaw, where the PD is most vulnerable [22]. Conversely, the most common place of the sialoliths in horses is within the PD, usually found at its most rostral portion, just before it opens into the vestibule against the maxillary third cheek tooth [17, 54]. Sialoliths can cause occlusion in the PD by forming salivary calculi, or stones. They may develop around a small foreign body that becomes lodged in the duct after entering through the oral cavity [52]. Sialolithiasis occurs more frequently in equine species than in other domestic species [17]. Based on our findings, no anatomical differences in the ductal macroanatomy of the donkey's PSG, when compared with the horse, were noted to have a possible influence on the higher predisposition of sialolith formation in donkeys.

#### 4.1.4. MSG Shape and Appearance

The MSG in herbivores exhibit variations in shape. In water buffaloes [32], cows [42], donkeys [35, 36], and horses [14, 31, 39, 40, 42], the MSG is curved or crescent-shaped. Nevertheless, it is triangular in dromedary [38], mouflon, and deer [37, 44], ovine, and caprine [31], or irregularly quadrilateral in ovine [45].

The MSG consists of a single portion in cows [31] and water buffaloes [32], two separable lobes in the mouflon, giraffes, and deer [37, 43, 44, 51], or four lobes in dromedaries [49]. In the current investigation, the MSG had two lobes, resembling the horse [31, 39]. Therefore, this finding appears to be a typical anatomical feature in domestic equid species.

#### 4.1.5. MSG Location and Relation

While the MSG in herbivores typically occupies the space between the basihyoid and the wing of the atlas [37, 38, 42], the MG displayed situation variations relative to the PSG and lower jaw among the species as follows.

In cows [31], ovine [45], and water buffaloes [32], as well as in horses [14, 23, 31, 41, 46], the MSG is laterad covered mainly by the PSG and partially by the mandibular angle. In the dromedary, the MSG is partially covered by the PSG, but its caudal and ventral edges extend beyond the PSG. In other words, the MSG is not covered by the lower jaw and is mostly positioned in the retromandibular fossa dromedary [38]. Nevertheless, in donkeys, the MSG is mostly covered by the ramus of the lower jaw. This suggests that the MG in the donkey tends to be situated more rostrad toward the lower jaw than in other reported animals, including horses, domestic ruminants, dromedaries, and water buffaloes.

The ventral extremity of the MSG is palpable in cows [14] and water buffaloes [32], but the MSG in donkeys is not reachable for palpation, resembling horses [14].

In contrast to the horse [14, 41, 46], the MSG in the donkey was not in association with the medial retropharyngeal lymph node.

The medial aspect of the horse MSG was in association with the external carotid artery [46] or common carotid artery [23, 31, 41, 42], while the donkey MG was not associated with the arteries.

#### 4.1.6. The Beginning, Route, and Ending of MD

Like the fallow deer [37], water buffaloes [32], and horses [14, 31, 39, 41, 42], the duct in donkeys is made by one radicle (branch). Nevertheless, in the cow [31], the duct is made by the joining of the dorsal and ventral radicles, and in the ovine [45], it is made by three or four radicles. In some cases, such as the mouflon and red deer [37], the duct is made of several radicles. The contribution of the radicles to forming a single major duct is a consistent anatomical feature in equine species.

As in the dromedary [49], horses [14, 31, 39, 41, 42], and cows [42], the duct in donkeys emerges from the rostral edge of the MSG. Nevertheless, in the water buffaloes [32], mouflon, and deer [37], the duct exits from the lateral aspect of the MSG, or in the case of the cow [31], it exits from the medial aspect of the MSG.

#### 4.1.7. SGs Existence

As in the dromedary [38] and horses [14, 31, 39, 41, 42], the monostomatic gland is absent in the examined donkeys. Nevertheless, in all wild and domestic ruminants, the SGs consists of monostomatic and polystomatic glands [14, 38, 43, 51]. Therefore, in contrast to ruminants, the lack of monostomatic SGs is a typical feature in equines and dromedaries. This absence seems to be compensated by the comparatively large and elongated polystomatic sublingual gland in these species. In wild and domestic ruminants, the polystomatic and monostomatic glands extend from the mandibular symphysis to the area near the cheek teeth.

#### 4.1.8. Polystomatic Sublingual Gland Location and Relation

The polystomatic SGs extends rostrad beyond the level of the first lower premolar tooth (P2) in red deer [38] and water buffaloes [32], or to the level of the third lower premolar tooth (P4) in fallow deer and mouflon [38], or to the incisive portion of the lower jaw in the horse, ovine, and cow [23, 31, 39, 42]. Thus, the rostral extent of the polystomatic SG in the donkey is at the level of the mandibular symphysis, resembling the horse [23].

The caudal extent of the polystomatic SGs in horses varies among the authors. In donkeys, the polystomatic SGs extend caudad to the level of the lower M2, resembling horses [14, 23, 39]. Nevertheless, in horses, the polystomatic SG continued to the level of the P4 [31, 40, 46] or M1 [23, 39, 42, 47] and to the rostral edge of the pterygoid muscle in red deer [37], beyond the palatoglossal arch in fallow deer, mouflon [37], and cows [42], or to the last lower molar tooth (M3) in ovine [45] and water buffaloes [32]. The polystomatic sublingual gland exhibits syntopic variations between the horse and the donkey. In the horse, the lingual nerve routed mediad to the polystomatic sublingual gland [31, 42, 46], while in the current investigation, the lingual nerve routed on the medial and lateral aspects of the polystomatic sublingual gland.

The monostomatic sublingual gland and lingual artery in the horse routed mediad throughout the polystomatic sublingual gland [47], while in the current investigation, the MD and lingual artery routed on the rostral half of this aspect near the dorsal edge.

The situation of the rostral and caudal extents of the polystomatic sublingual gland in donkeys resembled horses, dromedaries, and large domestic ruminants. Nonetheless, it was considerably different from that in wild and small domestic ruminants.

In summary, the general conformation of the MSGs' components in the donkey displays features that are highly displaced compared to the horse, regardless of the rostral situation of the MSG, the straight caudal edge of the PSG, the absence of perforation of the lateral aspect of the PSG by the maxillary vein, and the superficial situation of the PD at the level of the body of the lower jaw rostral to in front of the masseter muscle.

The general macroanatomy of the MSGs' components demonstrated marked variations and similarities among wild and domestic ruminants, *camelids* (dromedary, llama, and alpaca), and domestic equines (horse and donkey). The major variations consist of the route of the PD partially or completely filling the retromandibular space with PSG, the contribution of radicles to form the major MD and PD, the situation of the MSG relative to the lower jaw and its convergence by PSG, and the presence or absence of the monostomatic gland.

### 4.2. Sialoradiography

Sialoradiography, which resolves the intraglandular ductal structures, provides valuable information and aids in accurately diagnosing intraductal pathology. The technique helps differentiate between intact salivary glands and affected ones. Nevertheless, there is limited, scattered, and fragmentary information available in veterinary radiology textbooks [55, 56]. From the following viewpoints, we compare and discuss our results with the available literature on sialoradiography of animals.

#### 4.2.1. Contrast Mediums

As previously published data [26–30, 55] show, water-soluble media were utilized for sialoradiography in this investigation. Water-soluble media are ideal to remain within the ductal structures for a sufficient time. They also rapidly diffuse and dilute in saliva, providing clear and sharp ductal macroanatomy, similar to that achieved with lipid-soluble agents, although fat-soluble media may induce inflammatory reactions [21].

#### 4.2.2. Volume of the Contrast Medium

The dosage of the contrast media utilized for sialoradiography of the PD is 20 mL (in horse and cow) and 3–4 mL (in ovine and caprine), whereas the dosage of the contrast media for sialoradiography of MSG in horses is 20 mL, cows is 15 mL, and ovine and caprine is 2 mL. In water buffaloes [32], the total dose was 3–4.5 mL for the two phases. Nevertheless, in donkeys, 1 mL is utilized for the sialoradiography of the PSG or MSG. Accordingly, in this investigation, we utilized a small amount of the contrast solution to obtain ideal sialograms.

Based on current sialograms on the cow, horse, ovine, caprine, and dromedary [26–30], it is clear that authors administreted excessive contrast agent, leading to extensive acinarization of the glands and obscuring almost all the details of the intraglandular ductal structures. This has been regarded as one of the causes of sialogram failure. Therefore, only a small amount of contrast material should be injected for each phase.

#### 4.2.3. Radiographic Projection

In the available literature concerning sialoradiography in cows, ovine, caprine, horses, dromedaries, and water buffaloes, the lateral oblique projection of the head and proximal portion of the neck was made, similar to what was done for the donkey. Nevertheless, the ventrodorsal projection of the head was taken only in the dog's sialoradiography [55].

Superimposition of the salivary ductal structures occurs when bilateral sialoradiography is conducted in donkeys. Therefore, only a single sialogram can be made in donkeys, resembling those of cows, dromedaries, ovine, horses, caprine, and water buffaloes.

#### 4.2.4. Salivary Ductal Appearance

Although sialoradiography is the only modality to outline the fine branches of the intraglandular ductal structures, such accounts were ignored considerably in sialoradiography of the MSGs in cow, dromedary, ovine, horse, and caprine [26–30]. These studies primarily concentrated on outlining the shape and place of the MSGs and the single major duct. Nevertheless, the current investigation and our previous investigation [32] are the only reports on the sialoradiography of the animals that presented detailed and comprehensive data on the ductal structures.

As in the horses, cows, dromedaries, ovine, caprine, and water buffaloes, the major PD in donkeys appeared as a curve opening dorsally.

As in water buffaloes [32] and dromedaries [49], the intraglandular portion of the PD is made by two large radicles in the donkey. Tadjalli et al. [30], in caprine, documented that the duct was made by two large branches, but the opacification of the intraglandular structures was also clearly discernible on the sialograms. Furthermore, based on the parotid sialograms in cow, dromedary, and ovine [26–29], the intraglandular ductal structures were obscured due to excessive administration of the contrast medium.

As in water buffaloes [32], the intramandibular ductal structures are made by the confluence of several ductules in donkeys. It was composed of rostral and caudal radicles (branches) in cows, but their mandibular sialograms did not support such a description. Furthermore, the intramandibular ductal structures were obscured due to the excessive administration of the contrast medium in caprine [30], as well as ovine, dromedary, horses [26–29], and dromedary [49].

As in water buffaloes [32], the donkey's MD displayed a curvature (bending) opening dorsally. Nevertheless, the major MD in horses shows two or three angles, opening dorsad or ventrad [29].

Although cadaver CT sialography provides a three-dimensional visualization of the dog salivary glands and their duct [57], such a technique was not utilized in this investigation because the objective was to record sialograms made by the sialoradiography procedure.

In postmortem research, it is essential to understand how decomposition artifacts (e.g., degeneration of tissues and gas formation) can affect radiologic images [55, 56]. In this investigation, these artifacts were limited because of the usage of extremely fresh conditions of isolated heads.

On the other hand, the sialoradiography technique and appearance of the parotid and mandibular ductal structures did not differ between cadaver specimens and live animals [26–30]. Furthermore, they believed that performing sialoradiography in cadaver heads facilitates the catheterization of the salivary ducts. Therefore, the images and data reported in the current investigation may be utilized as basic information for veterinary clinicians, surgeons, and radiologists for (1) diagnosis of MSGs pathologies and (2) aiding in performing sialography in live donkeys.

## 5. Conclusion

The technique of cannulation, contrast media injection, and positioning for the sialoradiography in the donkey was thoroughly described. This is the first investigation to outline a high level of detail regarding the typical macroanatomy of the extra- and intraglandular ductal structures in lateral mandibular and parotid sialograms. The major salivary ductal structures and their glands' macroanatomy in the donkeys were similar to those in horses, regardless of the rostral situation of the MSG, the straight caudal edge of the PSG, the absence of perforation of the lateral aspect and gland substance of the PSG by the maxillary vein, the superficial situation of the PD on the body of the lower jaw rostral to in front of the masseter muscle, the situation of the PD opening against the crowns of the third and fourth premolar teeth, and the formation of the single major PD at the rostrolateral aspect of the dorsal portion of the PSG. The images and data reported in the current investigation may be utilized as basic information for veterinary clinicians, surgeons, and radiologists for (1) diagnosis of the MSGs pathologies and (2) aiding in performing sialography in live donkeys.

## Data Availability

The datasets generated during and/or analyzed during the current investigation are available from the corresponding author on reasonable request.

## Nomenclature

MSGs: Major salivary glands
MD: Mandibular duct
MSG: Mandibular salivary gland
PD: Parotid duct
SGs: Sublingual salivary glands
